# Catalytic Degradation of Organic Dyes Indicates Anti-Proliferative Effects of Magnetoelectric Nanoparticles

**DOI:** 10.1007/s11664-025-11843-5

**Published:** 2025-03-11

**Authors:** Max Shotbolt, Emily Zhu, Victoria Andre, Elric Zhang, Isabelle Duran, John Bryant, Wael El-Rifai, Ping Liang, Sakhrat Khizroev

**Affiliations:** 1 University of Miami, McArthur Engineering Building, Memorial Dr, Coral Gables, FL 33146 USA; 2 Miami Palmetto Highschool, 7431 SW 120th St, Pinecrest, FL 33156 USA; 3 MOFFITT Cancer Center, 603 N Flamingo Rd # 151, Pembroke Pines, FL 33028 USA; 4 Cellular Nanomed, Irvine at 8 Corporate Park, Irvine, CA 93606 USA

**Keywords:** Magnetoelectric, nanoparticles, catalysis, cancer therapeutics

## Abstract

Over the past decade, magnetoelectric nanoparticles (MENPs) have proven effective in generating local electric fields in response to stimulation with a magnetic field. The applications of such nanoparticles are many and varied, with examples of prior research including use for on-demand drug release, wireless modulation and recording of neural activity, and organic dye degradation. This study investigates the potential for organic dye degradation to be used as a rapid and efficient screening tool to detect the magnetoelectric effect of MENPs, and how the results of such a test mirror the antiproliferative effect of said nanoparticles. Trypan blue was selected as an azo dye to test for dye degradation. Vials of the dye were treated with CoFe2O4@BaTiO3 core-shell MENPs of varying characteristics, both with and without concurrent 1-kHz 250-Oe magnetic stimulation. Dye degradation was measured using ultraviolet (UV)-vis spectroscopy. Dye degradation efficacy varied with varying nanoparticle synthesis parameters. As controls, nanoparticles of the same composition, but with an insignificant magnetoelectric effect, were used. SKOV-3 ovarian cancer cells were then treated with the same nanoparticles, and viability was measured with an adenosine triphosphate (ATP) assay. These measurements show a decrease in cell viability up to 60.3% of control (*p* = 0.0052), which mirrored the efficacy of dye degradation of up to 69.8% (*p* = 0.0037) in each of the particle variants, demonstrating the value of azo dye degradation as a simple screening test for MENPs, and showing the potential of MENPs used as wirelessly controlled nanodevices to allow targeted electric field-based treatments.

## Introduction

The magnetoelectric (ME) effect has the potential to be a valuable tool for interacting with the body’s various electrophysiological systems.^1–7^ The ability to convert magnetic fields, which can pass through biological tissues with minimal effect, into highly localized electric fields offers the potential to interact with voltage gated ion channels, manipulate ion concentrations in and around cells, and, if necessary, rupture cell membranes through electroporation on demand, all non-invasively. Magnetoelectric nanoparticles (MENPs) can allow novel solutions to address some of the most pressing issues in medicine, such as on-demand, localized drug delivery, non-invasive deep brain stimulation, and targeted cancer therapeutics.^8^ MENPs are able to generate highly localized electric fields through the conversion of magnetic fields to electric fields. The core–shell design used in this study operates on the principle of a magnetostrictive core, which generates strain when exposed to an AC magnetic field, transferring this strain to a piezoelectric shell, which, in turn, generates electric fields. This allows for the wireless activation of MENPs and thus the generation of electric fields within the body without requiring the use of electrodes passing a voltage across tissue.

**Fig. 1:**
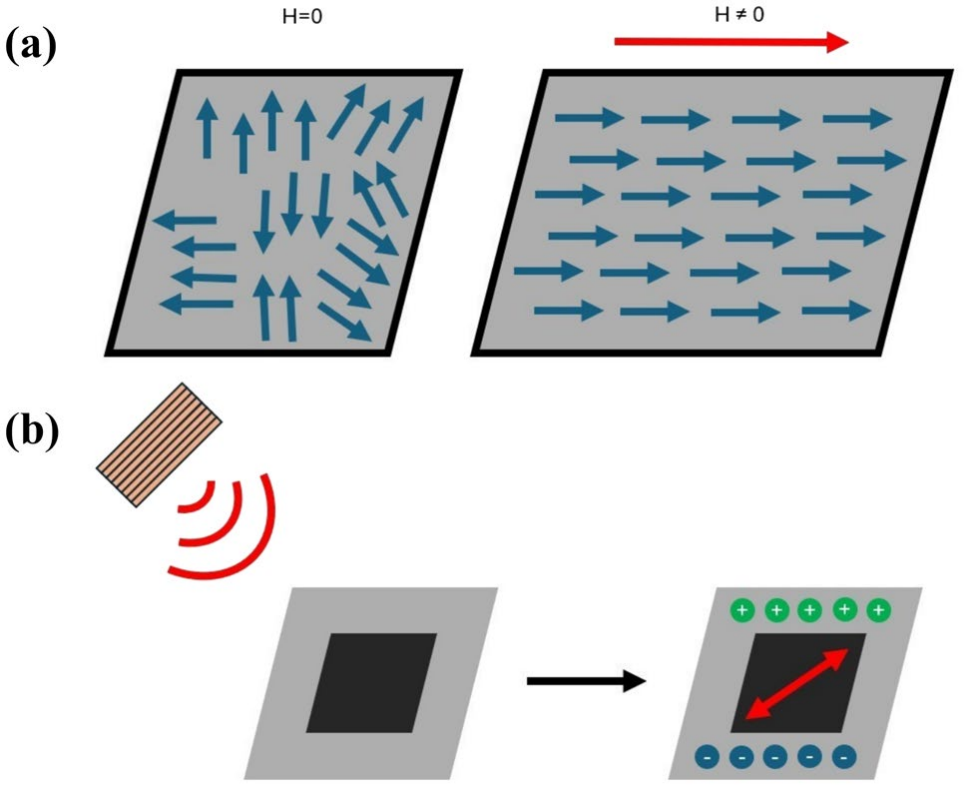
(A) The mechanism of magnetostriction. The alignment of otherwise disorganized magnetic domains creates a strain in the direction of a sufficiently large externally applied magnetic field. (B) The ME effect as achieved by MENPs. MENPs, when stimulated by an external magnetic field, generate local electric fields through the magnetostrictive core applying strain to the piezoelectric shell.

One of the most compelling applications of MENPs is in the development of wirelessly controlled targeted non-invasive electric field-based cancer treatments, such as irreversible electroporation (IRE) and tumor treatment fields.^9–15^ IRE is a technique used to ablate solid tumors, and potentially trigger an immune response, with adequate specificity^15–19^; however, current technology requires the application of high voltage through biological tissue, which carries significant risks, including potential damage to the arteries^20^ (Fig. 1).

MENPs may offer a promising high-specificity alternative, capable of generating electric fields, localized to tumor cells, and sparing healthy cells, sufficient to induce IRE without the need for invasive probes or the risks associated with passing currents through relatively large tissue regions. Additional complications of traditional IRE come from limitations in the volume of the ablated area.^21^ At present, to enlarge the scope of tumor treatment, the energy delivered must be amplified either by increasing the pulse amplitude, duration, number, or frequency, and, consequently, a larger ablation is associated with increased tissue heating, thermal damage, and diselectrolytemia, ^22^ or by using strategically placed electrode arrays or multiple treatments to ensure that the critical electric field encompasses the entire tumor. This method, however, requires additional treatment planning and electrode guidance for large tumor volumes. In contrast, if placed directly on the cancer cell membrane, MENPs can generate significantly higher electric fields across it (˜1000 V/cm),^23^ compared to the induced field in the traditional IRE setting (generating ˜1 V/cm across the membrane for the voltage of ˜1000 V applied between two electrodes with a 1-cm separation), while not affecting any surrounding healthy cells. As such, due to their highly localized treatment effects, MENPs may offer an alternative, in particular for larger tumor ablations. The large quantitative difference between the two cases is due to the conductivity difference between the conductive intra-/extra-cellular space and the dielectric membrane. Additionally, by dispersing MENPs across the tumor site, it may become possible to generate sufficient electric fields throughout the tumor without requiring a current across the site, as each particle independently generates a field.

**Fig. 2:**
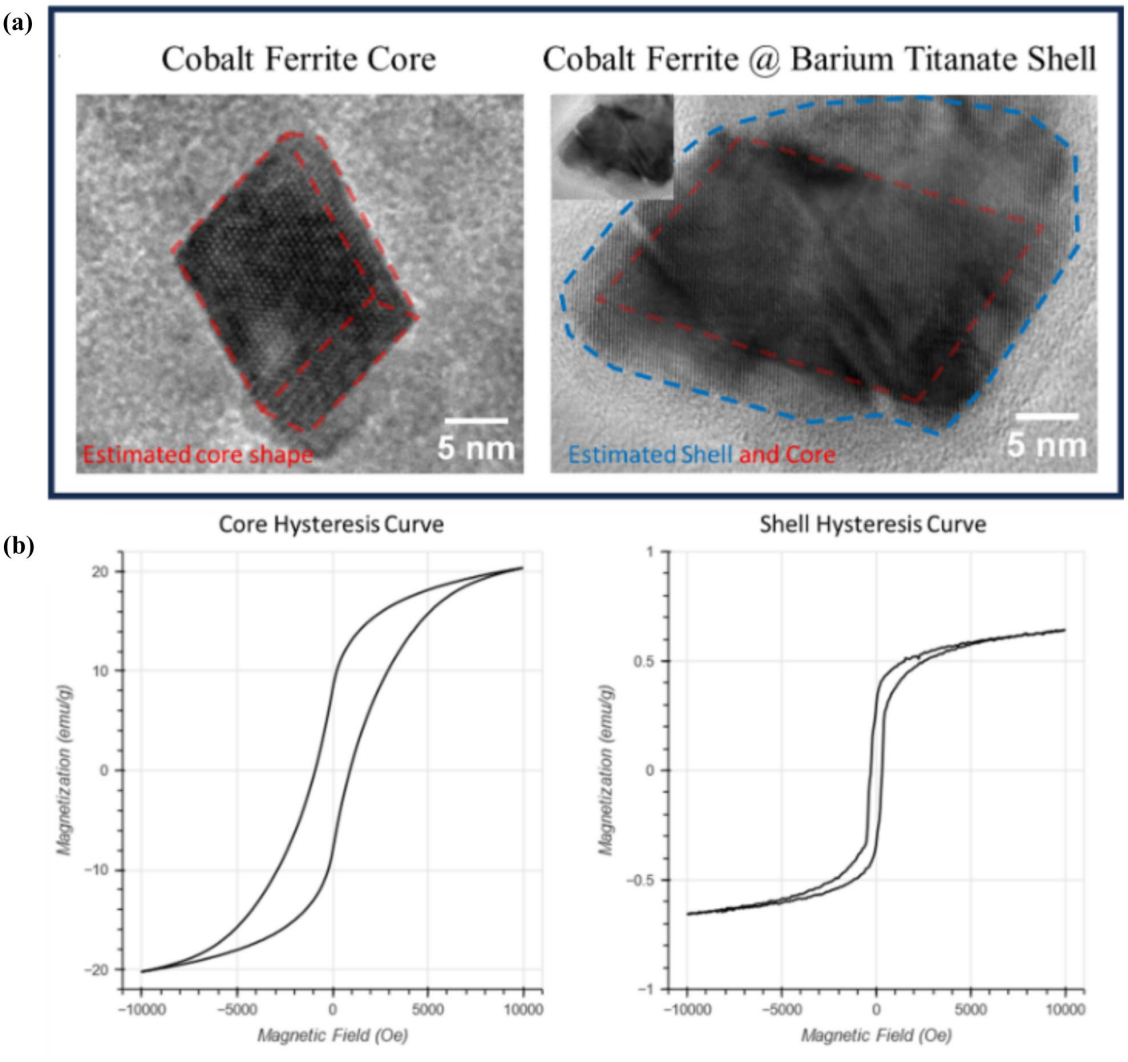
Structural characterization of MENPs: (A) high-resolution transmission electron microscopy (TEM) image showing the core-shell structure of MENPs, and (B) hysteresis curves of MENPs showing coercivity and saturation magnetization of particles.

The effectiveness of MENPs in this capacity is contingent upon their ability to produce strong electric fields. Previous iterations of MENPs have shown promise in generating relatively weak fields, sufficient to stimulate neurons and release drugs on command.^24–34^ However, to produce an IRE effect, it is necessary to improve the particles’ ME effect by several orders of magnitude.^23,35^ Currently, the most widely accepted MENPs make use of a magnetostrictive core of cobalt ferrite and a piezoelectric shell of barium titanate.^36^ These are still the best available materials, as they demonstrate the strongest magnetostrictive and piezoelectric effects and adequate lattice-matched interfacial coupling between the magnetic core and the piezoelectric shell while maintaining bioavailability, unlike lead-based alternatives such as lead zirconate titanite.^37,38^ Without changing materials, it is necessary to improve fabrication techniques to ensure optimal crystallinity, lattice-matched core–shell interface, shape, and size distribution. This can be achieved through minor alterations in the numerous fabrication techniques used.^7^ However, in order to facilitate the rapid and reliable optimization of MENP fabrication, a simple and efficient screening test is essential. Existing testing methods, such as in vitro cell stimulation or piezo force microscopy, are either slow and subject to biological variability or prohibitively complex, expensive, and time-consuming. Additionally, most of the existing technologies designed for the measurement of the magnetoelectric effect are only practical at the micrometer scale or above.^39^ There remain very few methods by which the measurement of the magnetoelectric effect of nanoparticles can be achieved, with the only other existing method relying on piezo-force microscopy, a technique which is subject to noise from electrostatic excitation via the conductive cantilever.^40^ The degradation of organic dyes, triggered by the ME effect under magnetic stimulation, offers a promising alternative.^41^ This approach not only offers a potential future solution for water treatment applications but also provides a rapid and quantifiable measure of MENP effectiveness, which is crucial for the iterative development of these nanoparticles. As such, this represents a novel option for future development of more precise measurement tools of the magnetoelectric effect at the nanoscale (Fig. 2).

In this paper, we explore the use of MENPs for two distinct purposes: the degradation of trypan blue dye as a proxy for their ability to generate local electric fields, and their application in reducing the viability of SKOV-3 ovarian cancer cells through IRE. The results of these studies have the potential to significantly improve the future development of MENPs, which in turn will improve their potential use as an anticancer therapeutic tool, demonstrating the versatility and promise of MENPs in medical applications.

## Experimental

### MENP Synthesis

Cobalt(II) nitrate hexahydrate(Co(NO_3_)2·6H_2_O), iron(III) nitrate nonahydrate (Fe(NO_3_)_3_·9H_2_O), sodium hydroxide (NaOH), barium carbonate (BaCO_3_), titanium(IV) isoproproxide (Ti[OCH(CH_3_)_2_]_4_), citric acid (HOC(COOH)(CH_2_COOH)_2_), polyethylene glycol (H(OCH_2_CH_2_)nOH, MW: 3000), and ethanol (99.7%), were purchased from Sigma Aldrich, Fisher Scientific, and Millipore Sigma. All the reagents were used without further purification.

The CoFe_2_O_4_@BaTiO_3_ core–shell nanoparticles were made in a two-step process. The CoFe cores were produced using the following co-precipitation process. In a typical synthesis, 100 mg of cobalt nitrate and 278 mg of iron nitrate were dissolved separately in DI water under constant 500-rpm stirring. After the metallic salts had fully dissolved, the solutions were combined and 3 M NaOH aqueous solution was added dropwise until the mixture reached the desired pH. The solution was then heated to 90 °C and allowed to react for a given time.

In order to vary the characteristics of the nanoparticles, two pH points were used for two different time points. A batch of MENPs with little to no core–shell interface was made using a pH of 11, which was allowed to react for 30 min. Two additional batches, pre-strained MENPs and MENPs with a strong core–shell interface, were prepared at a pH of 13 and allowed to react for 60 min, since a high pH favors particle nucleation rather than growth, thus requiring a longer reaction time.^42^

The solution was then cooled, and the CoFe particles separated from the solution using a permanent magnet. Subsequently, the particles were washed twice with DI water and once with pure ethanol before being left to dry overnight on a hotplate at 90 °C. The following day, the temperature was increased to 140 °C to ensure the dryness of the cores. After a minimum of an hour at this temperature, the cores were ground with a mortar and pestle until they reached a fine powder-like consistency.

In the next step, barium titanate shells were formed around these cores at a 1:3 ratio, using a modified sol–gel process.^43^ The precursors were prepared in three beakers. The first beaker contained 20 mg of dried CoFe cores with 1000 mg of citric acid in 20 ml of DI water, which was sonicated in a QSonica probe sonicator for 2 h at 3 s on/3 s off, at 20% amplitude. In order to achieve pre-strained particles, one of the batches of cores was magnetized by being placed on a strong permanent magnet, experiencing a field of approximately 1500 Oe for 30 min prior to sonication. The second beaker contained 76 l of titanium isopropoxide with 1000 mg of citric acid in 20 ml of ethanol, and the third beaker had 53 mg of barium carbonate dissolved with 1000 mg of citric acid in 20 ml of DI water. The second and third solutions were stirred at 500 rpm separately for an hour to ensure chelation before they were combined.

The solution was then brought to 90 °C and allowed to evaporate under constant 500-rpm stirring until it had been reduced to 20 ml. The cores were then added to the combined solution and reduced under constant heat and stirring to form the gel. This gel was transferred to an alumina crucible and calcined according to the following temperature profile: 25–800 °C ramp in 420 min, held at 800 °C for 6 h, and then cooled from 800 to 25 °C in 540 min. Once the particles had returned to room temperature, they were lightly ground using a mortar and pestle, forming a soft powder. The finished MENPs were then washed twice in DI water and twice in ethanol before being left to dry overnight on a 90-°C hotplate. The dried MENPs were characterized using various techniques, such as scanning electron microscopy and AGM to generate hysteresis curves.

### Pegylation of MENPs

To PEGylate the MENPs, the particles were dispersed 1:1 particle to DI water ratio for 2 h using a probe sonicator at 3 s on/3 s off at 20% amplitude. Polyethylene glycol (PEG) with a molecular weight of 1500 was added at 8% of the particle mass, and the solution was sonicated for an additional 2 h at the same parameters. Subsequently, the conjugated particles were removed and washed in DI water twice to remove excess PEG, before being redispersed in DI water.

### Dye Degradation Screening

**Fig. 3:**
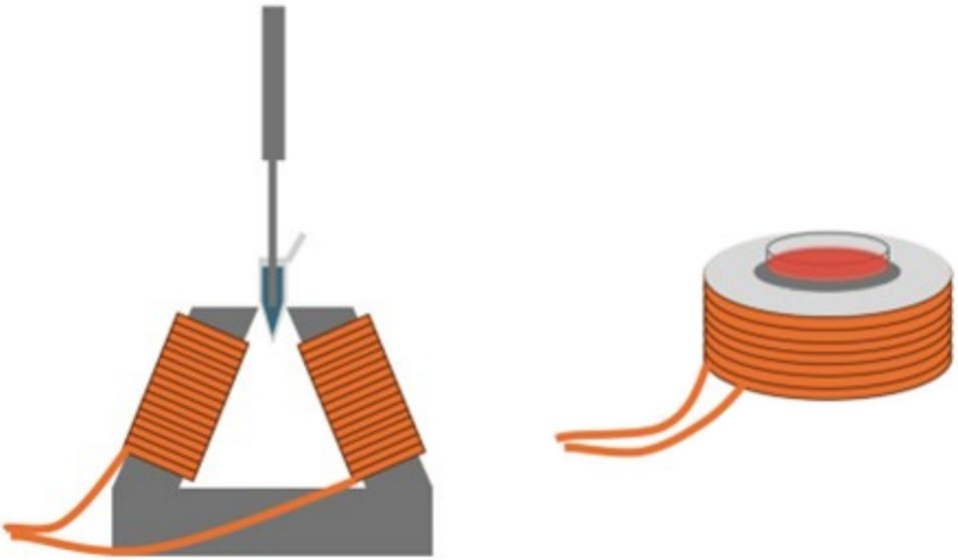
Experimental setup for dye degradation and in vitro anti-proliferation experiments: *left* For dye degradation studies, dye samples are mounted between two coils for continuous magnetic stimulation and simultaneous sonication; *right* for in vitro studies, cell dishes are placed over a coil for magnetic stimulation after MENP treatment.

First, a trypan blue stock solution was created by diluting trypan blue solution 0.4% (ThermoFischer) with DI water to a final concentration of 0.04%. Next, 5 mg of MENP were weighed and deposited in 1 ml of trypan blue stock solution and sonicated for 1 h to disperse the MENPs in each sample and to reach an adsorption/desorption equilibrium. These samples were split into 2 groups of 5 repeats: sonication, and magnetic stimulation. The sonication group samples were probe sonicated for an hour. The magnetic stimulation samples were probe sonicated for an hour while being stimulated by a 1-kHz 250-Oe magnetic field (Fig. 3). This was repeated for three different MENP types, each with minor fabrication differences. Additionally, a CoFe_2_O_4_ particle was used as a control, in order to account for any possibility of a Fenton-like reaction, which has been shown to degrade organic dyes through photo-catalysis.

After each sample completed its respective treatment, it was centrifuged to remove the MENPs. The supernatants were further diluted with DI water in a 1:4 ratio and the absorbance spectra were measured using a UV-Vis spectrophotometer. The peak absorbance at ˜580 nm of each group was compared to a control sample of untreated trypan blue stock solution. Each test was repeated 5 times, and a t-test was used to verify a statistically significant effect between treatment groups and the control sample (*a* = 0.05).

### SKOV-3 In Vitro Treatment

Human ovarian carcinoma cell line, SKOV-3, was used for the cell culture experiments. Cells were obtained from American Type Culture Collection in Manassas, VA, USA, and were maintained in McCoy’s 5A medium (Life Technologies, NY, USA) supplemented with 10% fetal bovine serum (Sigma-Aldrich) and 1% penicillin-streptomycin (science-cell).

SKOV-3 cells were cultured in 24-well plate at a density of 2 × 10^5^cells per well, and separated into 4 groups: controls, magnetic stimulation, MENP treatment, and MENP treatment with concurrent magnetic stimulation. After 24 h of incubation at 37 °C, the relevant cell groups were treated with 50 *µ*l of MENP solution and exposed to a 30-min magnetic field treatment of 1000-Oe 20-ms pulses every 5 s. After treatment, the cells were incubated for 24 h. A CellTiter-Glo ATP Assay (Promega) was used to measure cell viability, according to the manufacturer's instructions. The luminescent signal was read using a FLUOstar OPTIMA plate reader (BMG Labtech). Each test was performed in triplicate and repeated across three passages. An ANOVA test was used to determine statistical significance between groups.

## Results

**Fig. 4:**
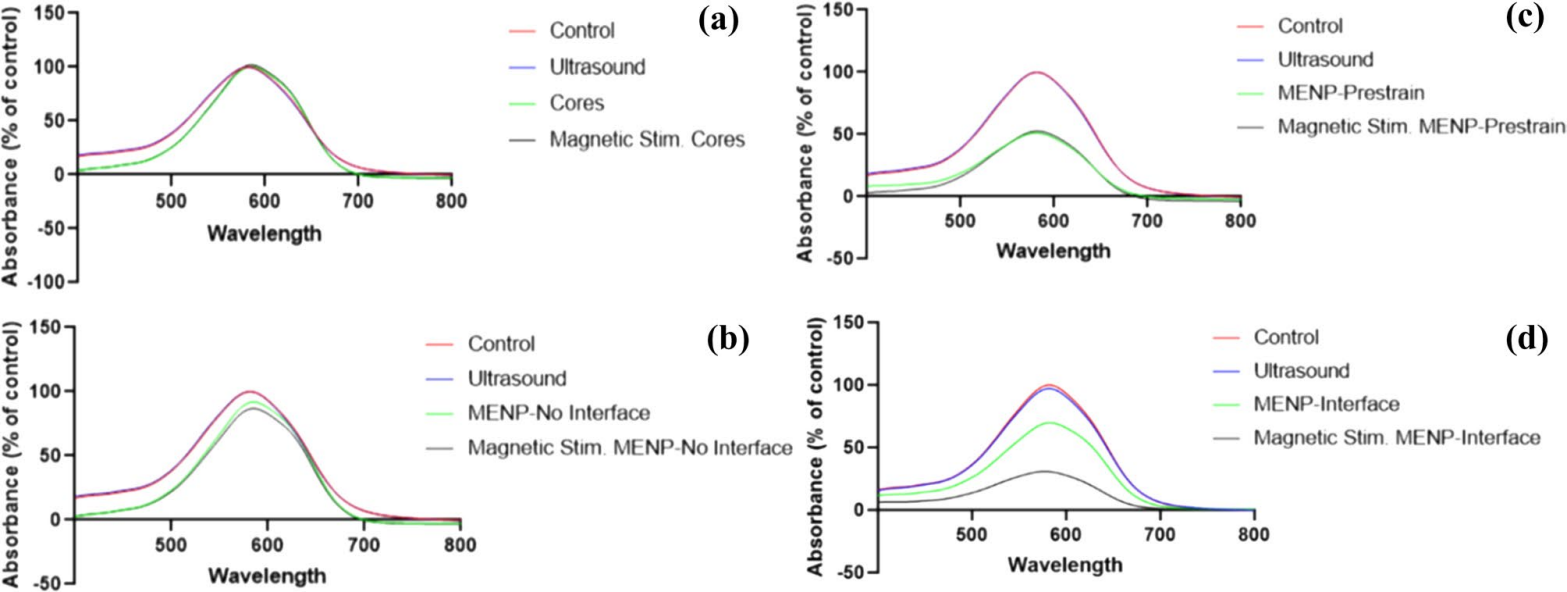
Absorbance spectra of trypan blue treated with (a) CoFe_2_O_4_ cores, (b) MENPs with no core–shell interface, (c) pre-strained MENPs, and (d) MENPs with core–shell interface both with and without concurrent magnetic stimulation.

By altering the MENP fabrication processes, a variety of effects could be achieved on the final dye concentration both with and without magnetic stimulation. Control particles made up of only CoFe_2_O_4_ showed little to no effect on the final dye concentration due to an absence of a significant surface charge on the particles regardless of magnetic stimulation, as shown in Fig. 4A.

Figure 4B shows the effects of MENPs with CoFe_2_O_4_ cores and BaTiO_3_ shells but with little lattice match between the core and the shell. The lack of any significant dye degradation demonstrates insufficient surface charge even when exposed to magnetic stimulation. This crucially demonstrates the sensitivity of the fabrication process even when the final chemical composition is identical, as it can completely remove any ME effect.

The pre-strained particles showed a significant decrease in the final dye concentration and no significant additional increase in effect when combined with magnetic stimulation, as shown in Fig. 4C. This is due to a long-term strain on the piezoelectric shell, which results in a surface charge that exists even when no magnetic stimulation is applied. This pre-strain effect is significantly greater than any additional strain that can be applied via the magnetoelectric effect, thus magnetic stimulation has no additional effect.

Figure 4D demonstrates that reducing the pre-existing strain on the MENP shell allows for a particle with little intrinsic effect on dye concentration, but, when combined with magnetic stimulation, generates an ME effect sufficient to degrade the dye solution.

**Fig. 5:**
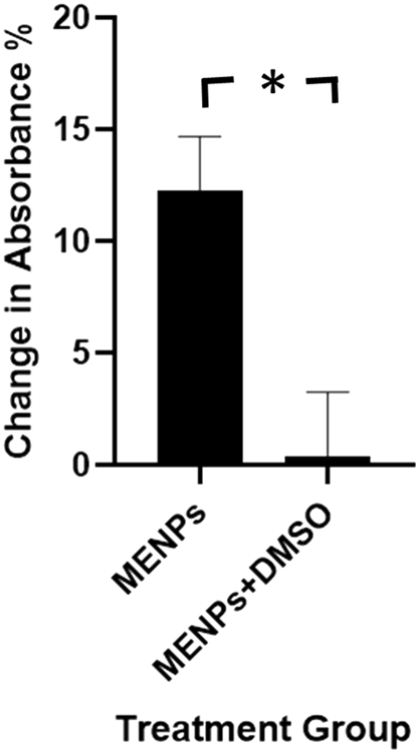
Reduction in peak absorbance of trypan blue treated with (a) MENPs with magnetic stimulation, (b) MENPs with magnetic stimulation and DMSO; *p* = 0.023.

In order to explore the mechanism of action behind the degradation of trypan blue dye, hydroxyl trapping experiments were performed. Dimethylsulfoxide (DMSO) was used as a hydroxyl scavenger. With the addition of 0.1 ml of DMSO, the dye degradation effect of MENPs could be nullified, even under identical stimulation parameters which had proven effective without the addition of DMSO, strongly corroborating the idea that the mechanism through which the ME effect degrades dye involves the generation of free radicals, hydroxyl radicals in particular, which then interact with the dye molecules (Fig. 5).

These results corroborate our hypothesis that MENPs interact with and degrade organic dyes via the generation of free radicals, It appears that MENPs are able to generate electric fields sufficient to create highly reactive radicals from water which, in turn, react with organic dye molecules.

### MENPs Generate Sufficient Electric Fields to Provide Anti-Cancer Effect on SKOV-3 Cells In Vitro

Particles with an insufficient core–shell interface, which had previously shown no significant dye degradation effects, did not show antiproliferative effects on SKOV-3 cells with or without magnetic stimulation.

The pre-strain particles showed a significant antiproliferative effect on SKOV-3, regardless of whether or not they were combined with magnetic field stimulation, of 55.34% (*p* = 0.0148) and 54.47% (*p* = 0.0012), respectively. This closely matches with the dye degradation results, demonstrating a strong intrinsic field effect but no increased effect during magnetic stimulation.

**Fig. 6:**
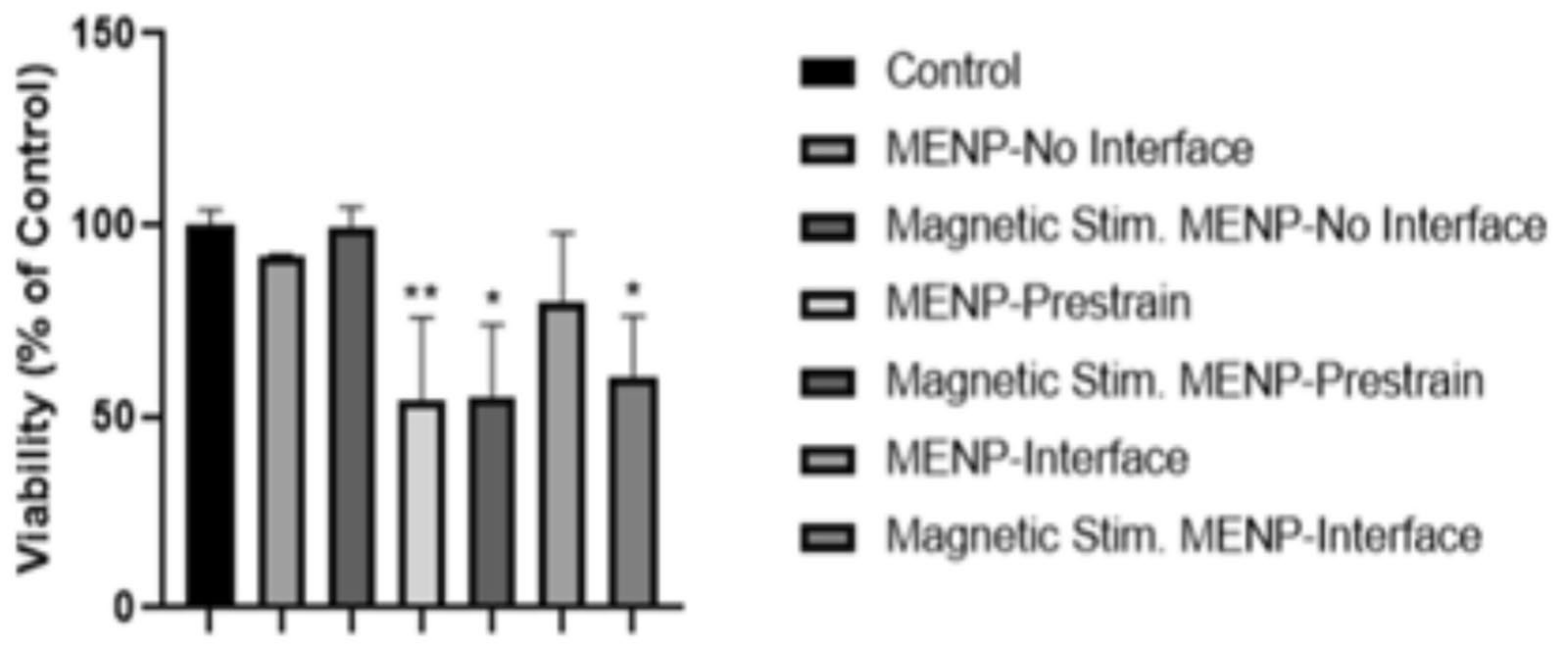
SKOV-3 cell viability 24 h after MENP treatment with MENPs with no core-shell interface, pre-strained MENPs, and MENPs with a core–shell interface both with and without concurrent magnetic stimulation.

Low-strain MENPs showed no significant impact on the viability of the SKOV-3 cells on their own. However, when MENPs were combined with magnetic field stimulation, viability decreased significantly to 60.3% of the control (*p* = 0.0052). This agrees once again with the expected results based on the dye-degradation screening test (Fig. 6).

## Discussion

Ovarian cancer is a significant health concern, being the deadliest gynecologic cancer^44^and the sixth leading cause of cancer-related deaths in women. The current standard of care for ovarian cancer involves a combination of debulking surgery and drug treatment, often including platinum-based chemotherapy. However, the prognosis for advanced ovarian cancer remains poor, with a high rate of recurrence, leading to the need for novel and targeted treatment options. Despite the emergence of new therapies, such as targeted agents and immunotherapy, the high rate of recurrence and the development of resistance to existing treatments present significant challenges.^45^ Additionally, the lack of efficient screening procedures for early detection further complicates the management of ovarian cancer. Therefore, there is a critical need for further research and the development of more advanced and effective treatment strategies to improve the outcomes for patients with ovarian cancer. MENPs may provide an additional tool in the future treatment of this and a variety of other cancers which present with solid tumors. This will only be achieved through the optimization of MENP fabrication, surface modification, and magnetic field stimulation techniques to enhance their therapeutic efficacy, all of which are dependent on a rapid testing technique for evaluating their performance.

**Fig. 7:**
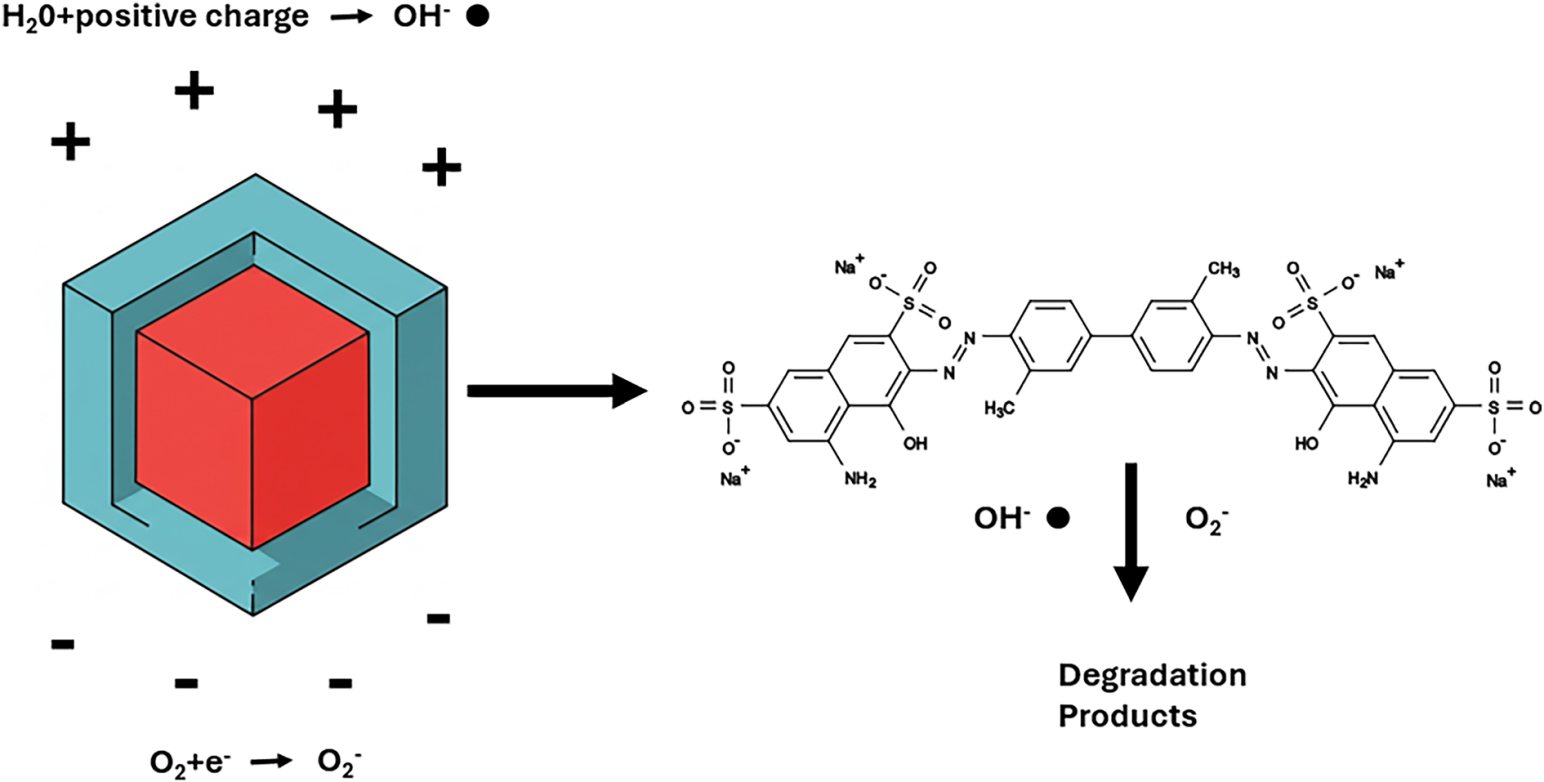
Dye degradation mechanism. Upon activation, MENPs generate electric fields which generate superoxide and hydroxyl radicals from oxygen and water. These reactive species react with the dye, degrading it.

The closeness with which the dye degradation test matches with the SKOV-3 anti-proliferative effect is indicative of the efficacy of the test. Given that this test can be completed within a few hours, at a very low cost, it presents a significant improvement on existing testing methods in these important metrics. While using the dye degradation test as a proxy for ME effect is not without limitations, it appears to be functional as a rapid screening tool in MENP development. Not only can dye degradation be used to indicate the generation of electric fields but it can also be used to quantify the strength of the ME effect in MENPs and to determine the conditions under which an electric field is generated. Current research indicates that, in similar particles, AC fields induce strain on the piezoelectric shell, polarizing the barium titanate to form surface charges. These charges then interact with surrounding oxygen and water to generate superoxide and hydroxyl radicals. These radicals can then interact with organic dye molecules, leading to their degradation.^41^ This is also corroborated by our results (Fig. 7).

Future research will be necessary to further elucidate the mechanisms by which the MENPs’ generated fields interact with cancer cells.

In particular, this research demonstrated the ability for dye degradation to predict the success of reducing the proliferation of SKOV-3 cancer cells under different stimulation conditions. The test was able to determine whether MENPs would generate an electric field both with and without the presence of an external magnetic field, which would then be verified by the in vitro results.

**Fig. 8:**
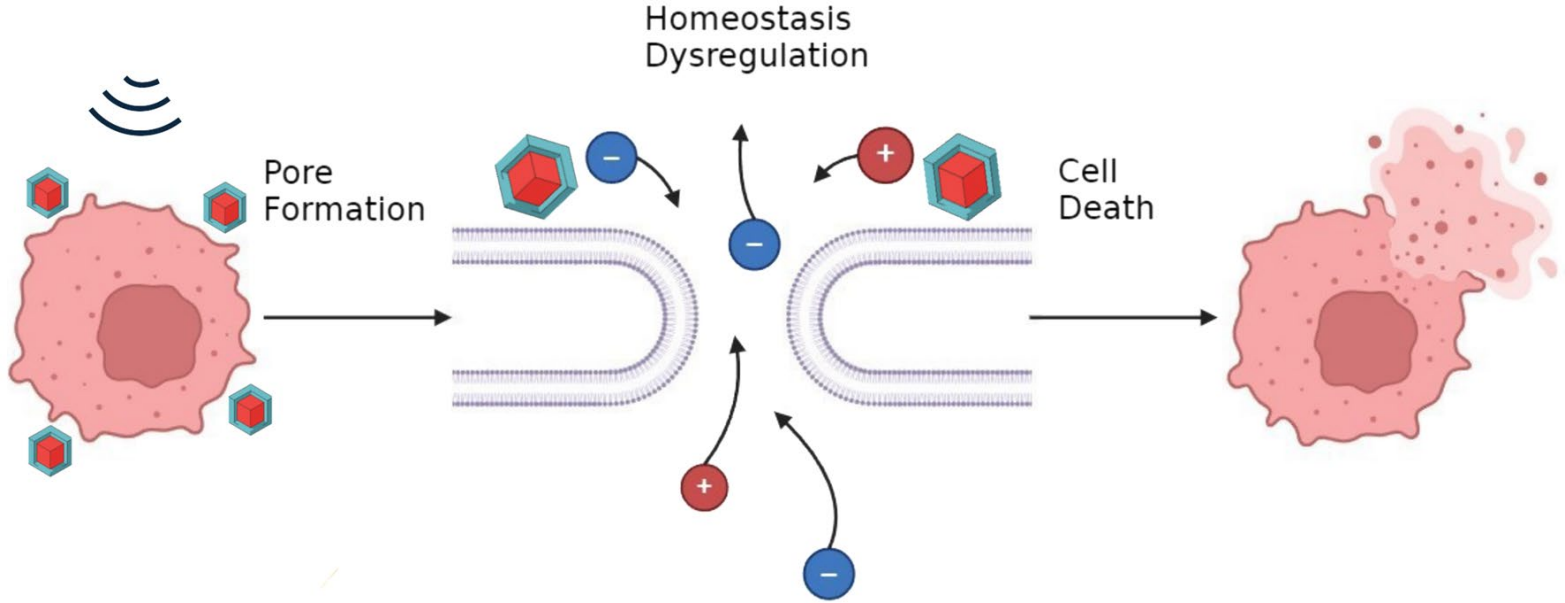
Proposed electroporation treatment effect of MENP-based IRE treatment. MENPs may be added into the tumor site and stimulated with an external magnetic field. This generates local electric fields sufficient to porate cell membranes. These pores disrupt the homeostatic equilibrium between the cell and the tumor micro-environment, leading to cell death.

The results of the ATP assay in SKOV-3 cells demonstrate the need for rapid testing and iteration, as, while the chemical composition of all MENPs tested were the same, even minor changes in the fabrication procedure resulted in a wide variety of outcomes on cell viability. An insufficient core–shell interface resulted in particles which had no significant impacts on cell viability. A strong core–shell interface could create a pre-strained shell and thus a long-term surface charge capable of causing damage to cells even without magnetic stimulation. This may be of some value in its own right, but it removes the on/off control possible with true magnetoelectrics. When the core–shell lattice interface is optimized to allow for magnetic control, it was possible to create particles which had insignificant inherent biological effects but were able to kill SKOV-3 cells when stimulated by an AC magnetic field (Fig. 8).

While it is highly likely that future iterations of MENPs will further enhance their effectiveness, this study demonstrates the possibility of MENPs killing cancer cells through electric field generation. With further optimization, this may open an avenue to improved IRE treatments for patients with nonresectable tumors that current techniques are not able to ablate.

## Conclusions

The significance of our findings lies not only in the immediate results but also in the broader implications for rapid screening and targeted cancer therapy. The ability of MENPs to degrade dyes up to 50% when exposed to a magnetic field serves as a rapid and cost-effective test for evaluating the effectiveness of MENPs. This is particularly important because dye degradation is a proxy for the MENP’s ability to generate local electric fields, which is a critical factor in their therapeutic potential. Dye degradation as a test is advantageous because it is faster and less variable than biological assays, which can be influenced by biological variability and are more time-consuming and expensive.

Furthermore, the potential importance of MENPs in IRE treatment cannot be overstated. The ability to reduce SKOV3 cell viability by over 50% with magnetic field exposure suggests a powerful application in oncology. MENPs that can be activated and deactivated by a magnetic field represent a significant advancement in cancer therapeutics because they offer a new targeting mechanism: particles can not only be targeted to a particular location but the activation field can itself be targeted to activate only MENPs in that region. This on-demand control could potentially minimize side effects and improve the therapeutic index of anticancer drugs by targeting and activating the particles precisely at the tumor site, thereby sparing healthy tissue.

The concept of using a magnetic field to control drug activity is a transformative approach to chemotherapy. It aligns with the principles of precision medicine, offering a method to localize treatment effects and reduce systemic toxicity. This could lead to a new class of therapeutics that are only active when and where they are needed, which is particularly valuable in the treatment of cancers where traditional chemotherapy can cause significant collateral damage to healthy cells.

While the only MENPs used for this study were cobalt ferrite-/barium titanate-based, and the only dye used was trypan blue, the applicability of dye degradation as a test for the efficacy of MENPs is broader than the particular setup used in this study. A similar study has been performed using methylene blue dye and bismuth ferrite-based MENPs, showing similar results.^41^ This is likely due to the mechanism of action. Rather than a direct interface between MENP and the dye molecules, the MENPs generate oxidative species in the solution, which, in turn, interacts with and degrades azo dyes. Given that there is no direct interaction between the dye and the MENP surface, it is likely that a number of piezoelectric shell materials would elicit similar effects, depending on their piezoelectric coefficients. For our purposes, barium titanate was chosen due to its bioavailability and high piezoelectric coefficient.

Future research will elucidate the optimal fabrication procedures to achieve the greatest magnetoelectric coefficient, without sacrificing the nanoscale. There are a variety of strategies to potentially improve current MENP fabrication, such as testing different piezoelectric shell and magnetostrictive core materials. Additionally, there is work to be done to optimize the core:shell mass ratio, maximizing the piezoelectric shell material which can still be driven by the magnetostrictive core. There is also the potential for testing a range of MENP volumes, depending on the desired outcomes (i.e., larger MENPs may generate stronger fields, but may not be suitable for penetrating the blood–brain barrier).

In summary, our study not only demonstrates the potential of MENPs in dye degradation and cancer cell viability reduction but also underscores the broader implications for rapid MENP screening and the development of innovative cancer therapies. The ability to activate and deactivate therapeutic effects with a highly targeted electric field controlled by a magnetic field could revolutionize the way we approach the treatment of cancer and other diseases where localized therapy is beneficial. Further research is needed to optimize MENP design and to fully understand the mechanisms behind their effects, paving the way for their clinical application.
